# Parental Smoking and Risk of Childhood Brain Tumors by Functional Polymorphisms in Polycyclic Aromatic Hydrocarbon Metabolism Genes

**DOI:** 10.1371/journal.pone.0079110

**Published:** 2013-11-18

**Authors:** Jessica L. Barrington-Trimis, Susan Searles Nielsen, Susan Preston-Martin, W. James Gauderman, Elizabeth A. Holly, Federico M. Farin, Beth A. Mueller, Roberta McKean-Cowdin

**Affiliations:** 1 Department of Preventive Medicine, University of Southern California, Keck School of Medicine, Los Angeles, California, United States of America; 2 Public Health Sciences Division, Fred Hutchinson Cancer Research Center, Seattle, Washington, United States of America; 3 Department of Epidemiology and Biostatistics, School of Medicine, University of California San Francisco, San Francisco, California, United States of America; 4 Functional Genomics Core Laboratory, Center for Ecogenetics and Environmental Health, University of Washington, Seattle, Washington, United States of America; 5 Department of Epidemiology, School of Public Health and Community Medicine, University of Washington, Seattle, Washington, United States of America; National Cancer Institute, National Institutes of Health, United States of America

## Abstract

**Background:**

A recent meta-analysis suggested an association between exposure to paternal smoking during pregnancy and childhood brain tumor risk, but no studies have evaluated whether this association differs by polymorphisms in genes that metabolize tobacco-smoke chemicals.

**Methods:**

We assessed 9 functional polymorphisms in 6 genes that affect the metabolism of polycyclic aromatic hydrocarbons (PAH) to evaluate potential interactions with parental smoking during pregnancy in a population-based case-control study of childhood brain tumors. Cases (N = 202) were ≤10 years old, diagnosed from 1984–1991 and identified in three Surveillance, Epidemiology, and End Results (SEER) registries in the western U.S. Controls in the same regions (N = 286) were frequency matched by age, sex, and study center. DNA for genotyping was obtained from archived newborn dried blood spots.

**Results:**

We found positive interaction odds ratios (ORs) for both maternal and paternal smoking during pregnancy, *EPHX1* H139R, and childhood brain tumors (*P*_interaction_ = 0.02; 0.10), such that children with the high-risk (greater PAH activation) genotype were at a higher risk of brain tumors relative to children with the low-risk genotype when exposed to tobacco smoke during pregnancy. A dose-response pattern for paternal smoking was observed among children with the *EPHX1* H139R high-risk genotype only (OR_no exposure_ = 1.0; OR_≤3__hours/day_ = 1.32, 95% CI: 0.52–3.34; OR_>3hours/day_ = 3.18, 95% CI: 0.92–11.0; *P*_trend_ = 0.07).

**Conclusion:**

Parental smoking during pregnancy may be a risk factor for childhood brain tumors among genetically susceptible children who more rapidly activate PAH in tobacco smoke.

## Introduction

The association between parental smoking during pregnancy and risk of childhood brain tumors is inconsistent in the literature. Most studies have reported positive associations between paternal smoking during pregnancy and childhood brain tumor risk, although the findings from only three studies were statistically significant [1]–[3]. Seven studies reported positive, but non-statistically significant associations [4]–[10], and two reported no association [11], [12]. A meta-analysis, combining ten studies published prior to 2000, estimated a 22% increase in risk of childhood brain tumors with exposure to paternal tobacco smoke during pregnancy (95% CI: 1.05, 1.40) [13].

Studies examining the association between maternal smoking during pregnancy and childhood brain tumors generally suggest little to no increased risk. Ten studies reported no association [1], [2], [5], [8], [10], [11], [14]–[17], and six studies reported a positive, but statistically non-significant association [4], [6], [9], [18]–[20]. Two meta-analyses estimated a statistically non-significant 4–5% increase in childhood brain tumor risk with maternal smoking during pregnancy using 12 of the above studies [13], [21]. However, a more recent prospective study reported a statistically significant 24% increase in childhood brain tumor risk with maternal smoking during pregnancy [22]. Although many studies have evaluated parental smoking and childhood brain tumors, none have evaluated potential interactions with functional polymorphisms in genes whose enzyme products metabolize tobacco smoke carcinogens, such as polycyclic aromatic hydrocarbons (PAH). Animal studies suggest this class of chemicals may possibly affect brain tumor risk [23], [24].

Several genes are associated with the activation (transformation to more carcinogenic intermediates) or detoxification of PAH. We focused on 6 genes of potential importance to our analysis of parental smoking (PAH exposure) and childhood brain tumors (Table 1). Microsomal epoxide hydrolase (mEH), coded by *EPHX1,* detoxifies selected substances (by catalyzing the hydrolysis of epoxide intermediates for excretion), and activates others, including PAH [25], [26]. Single nucleotide polymorphisms (SNPs) in exon 3 (Y113H) and exon 4 (H139R) of *EPHX1* alter enzyme activity through amino acid changes [25], [27]. A variant leading to a histidine (H) replacement of tyrosine (Y) at *EPHX1* Y113H results in decreased mEH activity, whereas a variant leading to an arginine (R) substitution of a histidine (H) at H139R results in increased mEH activity [27].

**Table 1:** Characteristics of Candidate Polymorphisms in Polycyclic Aromatic Hydrocarbon (PAH) Metabolism Genes.

Enzyme	Expression	Gene	Polymor. ID	Polymor.	Chr.	Enzyme Effect	Effect of High-Risk Allele	Ref.
Microsomal Epoxide Hydrolase (mEH)	*Fetus: Yes Brain:* *Yes* [38]	*EPHX1*	rs2234922	H139R	1	Activates PAHs	R: Faster PAH activation	[25]–[27]
		*EPHX1*	rs1051740	Y113H	1	Activates PAHs	Y: Faster PAH activation	
		*EPHX1*	rs2854448	C-613T	1	Activates PAHs	T: More mEH (faster PAH activation)	
Myeloperoxidase	*Brain: Yes* [46]	*MPO*	rs2333227	G-463A	17	Activates PAHs	G: Greater activity (faster PAH activation)	[28]
Sulfotransferase 1A1	*Fetus: Yes Brain:* *Yes* [47]	*SULT1A1*	rs9282861	R213H	16	Activates PAHs	R: Greater activity (faster PAH activation)	[29]
NAD(P)H: Quinone Oxireductase	*Brain: Yes* [48]	*NQO1*	rs1800566	P187S	16	Catalyzes detoxification ofPAH quinines	S: Reduced enzyme activity(reduced PAH detoxification)	[30], [31]
GlutathioneS-Transferase Pi 1	*Fetus: Yes Brain:* *Yes* [49]	*GSTP1*	rs1695	I105V	11	Detoxifies PAH intermediates	V: Reduced PAH detoxification	[32]–[34]
		*GSTP1*	rs1138272	A114V	11	Detoxifies PAH intermediates	V: Reduced PAH detoxification	
GlutathioneS-Transferase Mu 1	*Brain: Yes* [49]	*GSTM1*		Null	1	PAH detoxification	Null: No enzyme activity(reduced PAH detoxification)	[32]–[34]

Myeloperoxidase (*MPO*) and sulfotransferase (*SULT1A1*) also activate carcinogens found in tobacco smoke, including PAHs. Variations in genotype at *MPO* G-463A [28], or *SULT1A1* R213H [29] result in greater enzyme activity leading to faster PAH activation. NAD(P)H: quinone oxidoreductase (*NQO1*), and glutathione S-transferases (including *GSTM1* and *GSTP1*) detoxify PAHs. Variant alleles at *NQO1* (P187S) [30], [31], *GSTP1* I105V and *GSTP1* A114V [32]–[34], or a null genotype at *GSTM1* [34] result in decreased enzyme activity (detoxification) of at least some PAHs.

We analyzed the interaction between childhood brain tumors, exposure to parental smoking during pregnancy, and the child’s genotype for the above 9 functional polymorphisms to evaluate whether the association between childhood brain tumors and parental smoking during pregnancy varies by genetic polymorphisms in the child.

## Materials and Methods

### Participants

Participants were cases and controls enrolled in the West Coast Childhood Brain Tumor study [35] for whom a dried blood spot was located in newborn screening archives in California or Washington state (202 cases/286 controls) [36]. Cases were identified through the Surveillance, Epidemiology and End Results (SEER) registries in the Los Angeles, San Francisco-Oakland, and Seattle regions, and include children diagnosed with a tumor of the brain, cranial nerves, or meninges [*International Classification of Diseases-Oncology* (ICD-O) (World Health Organization 1976) codes 191.0–192.1] between 1984–1991. Controls living in the same regions were identified using random digit dialing, and were frequency matched to cases by age, sex, and study center. This analysis includes children born in Washington State in 1978 or later, or in California in 1982 or later, the birth years for which a specimen could still remain in the state archives. Children meeting these criteria were ≤10 years old. Specimens were obtained for 93% of eligible cases and 83% of eligible controls, as detailed elsewhere [36]. Cases and controls in this sample were similar to those in the larger study with respect to race/ethnicity and maternal education, but were born more recently and were therefore younger at diagnosis/reference date. Fewer astroglial cases and more medulloblastoma/primitive neuroectodermal tumor (PNET) cases were included in the present sample, consistent with a younger age at diagnosis [36]. Fewer case and control mothers and fathers smoked during pregnancy in more recent years than during earlier years.

### Exposure to Parental Smoking

Parental smoking was assessed by in-person interview with the subjects’ mothers. Mothers were asked if they ever smoked tobacco during their pregnancy with the enrolled child (yes/no), and the number of cigarettes smoked per day or week. They also were asked whether there was regular tobacco smoke exposure during pregnancy (yes/no, and hours per day) from the child’s father in the home, from any other household resident, or at work. Maternal exposure to tobacco smoke from the child’s father during pregnancy will be hereafter referred to as “paternal smoking.” Mothers and fathers also were asked if they ever smoked at least once a day for 3 months or longer prior to the pregnancy with the participating child (yes/no).

Maternal smoking during pregnancy was categorized by the typical number of cigarettes smoked per day: never smoked, 1–10, or 11+ cigarettes. Paternal smoking during pregnancy was categorized by the median number of hours per day the mother was exposed to tobacco smoke from the father (none, ≤3 hours per day, >3 hours per day).

### Genotyping

Subjects’ DNA was extracted from dried blood spot specimens from neonatal screening archives in California and Washington using the QIAamp DNA Mini Kit (QIAGEN, Valencia, CA) at the Center for Ecogenetics and Environmental Health Functional Genomics Laboratory at the University of Washington (Seattle, WA). Custom TaqMan Detection System-based assays-by-Design Service (Applied Biosystems, Inc., Foster City, CA) were used to assess *EPHX1* H139R (rs2234922), *EPHX1* Y113H (rs1051740), and *EPHX1* C-613T (rs2854448), *SULT1A1* R213H (rs9282861), *NQO1* P187S (rs1800566), *GSTP1* I105V (rs1695), *GSTP1* A114V (rs1138272), and rs2243828 (in complete linkage disequilibrium with *MPO* G-463A (rs2333227)). Microsomal epoxide hydrolase (mEH) activity was computed using *EPHX1* H139R and Y113H polymorphisms: low activity–0,1, or 2 stable alleles at H139R/Y113H (HH/HH, HH/HR, HY/HH, HH/RR, HY/HR, YY/HH), or high activity–3 or 4 stable alleles (HY/RR, YY/HR, YY/RR). One multiplex PCR-based assay [37] assessed GSTM1 null status. Complete genotyping data for all 9 polymorphisms was available for 200 (99.0%) cases and 284 (99.6%) controls. For 6% of cases and controls, duplicate and quadruplicate specimens were analyzed, blinded to initial results; analyses demonstrated complete concordance. Hardy Weinberg equilibrium was met (*P*>0.01) for all genotype frequencies for controls when stratified by race/ethnicity, with the exception of *EPHX1* Y113H for Los Angeles non-Hispanic Whites (*P*<0.0001), and for *NQ01* P187S for the heterogeneous ‘Other’ ethnicity (*P* = 0.0003).

### Statistical Analysis

We used unconditional logistic regression to evaluate the primary associations and potential interaction of genotype at each locus with maternal and/or paternal smoking during pregnancy. Odds ratios (ORs) and 95% confidence intervals (CIs) were computed to estimate relative risks. For main associations and interaction analyses, genotypes were dichotomized and classified as low- or high-risk based on the ability of each variant to increase or decrease the activation or detoxification of PAHs (Table 1). All models were adjusted for frequency matching factors (age at diagnosis/reference age (<5, 5–10 years), sex, region (Los Angeles, San Francisco, Seattle), race/ethnicity (African-American, Non-Hispanic White, Hispanic, Asian/Other), and birth year (1978–84, 1985–90)). Models were also adjusted for mother’s education (no college, some college, college or graduate degree) *a priori* with the expectation that maternal education is associated both with maternal or paternal smoking and childhood brain tumors. A parallel set of models were additionally adjusted for spousal smoking. Formal tests of interaction were conducted using a product term in each model. Case-only analyses were conducted after confirming independence of each gene-smoking association among controls. Consistencies of all associations were further evaluated by race/ethnicity (non-Hispanic White or Hispanic). Polytomous logistic regression was used to evaluate whether gene-environment interactions differed by histological tumor type (astroglial, medulloblastoma/PNET, or ependymoma/other); formal tests of heterogeneity were conducted. Tests for trend in dose analyses were evaluated using a 1df test for the categorized dose variable. Due to *a priori* hypotheses regarding the suspected functionality of the tested polymorphisms in the metabolism of tobacco smoke, no corrections for multiple comparisons were made. All reported *P-*values are two-sided.

### Ethics Statement

Institutional Review Board approvals were obtained in California from the University of Southern California Institutional Review Board and the Committee for the Protection of Human Subjects at the Health and Human Services Agency of the State of California, and in Washington from the Fred Hutchinson Cancer Research Center and the Washington State Department of Health. Written informed consent for all participants was obtained prior to interview. Before release from neonatal archives in both California and Washington, all dried blood-spot specimens were anonymized by the assignment of a random specimen identification number that could not be linked to identifying information.

## Results

Cases and controls were similar with regard to frequency-matched variables (Table 2). A higher proportion of controls were white (67.8% v. 53.6%, *P* = 0.02), and control mothers were more likely to have a college or graduate degree (29.8% vs. 20.8%, *P* = 0.02).

**Table 2:** Demographic Characteristics of Children With and Without Brain Tumors, West Coast Childhood Brain Tumor Study, Born 1978–1990.

		Cases n(%)	Controls n(%)
		N = 202	N = 285
Race/Ethnicity			
	White	105 (53.6)	192 (67.8)
	Hispanic	62 (31.6)	61 (21.6)
	African American	14 (7.1)	13 (4.6)
	Asian/other	15 (7.7)	17 (6.0)
	Unknown	6	2
Male		121 (59.9)	168 (58.9)
Birth year			
	1978–1980	10 (5.0)	27 (9.5)
	1981–1983	52 (25.7)	80 (28.1)
	1984–1986	93 (46.0)	107 (37.5)
	1987–1990	47 (23.3)	71 (24.9)
Age at diagnosis (years)^a^			
	<5	168 (83.2)	222 (77.9)
	5–10	34 (16.8)	63 (22.1)
Mother’s Education			
	No college^b^	103 (51.0)	112 (39.3)
	Some college (no degree)	57 (28.2)	88 (31.9)
	College or graduate degree	42 (20.8)	85 (29.8)
Histologic tumor type			
	Astroglial	97 (48.0)	
	PNET^c^	55 (27.2)	
	Other	50 (24.8)	

a Reference age for controls.

b <High school degree, high school degree, or basic or technical training only.

c Primitive neuroectodermal tumor.

The ORs for childhood brain tumors in relation to maternal smoking during pregnancy were less than one, but not statistically significant (Table 3). One exception was maternal smoking at the lowest smoking level (OR = 0.23; 95% CI: 0.08, 0.65) relative to never smoking.

**Table 3:** Risk of Childhood Brain Tumors in Relation to Exposure to Parental Smoking during pregnancy, West Coast Childhood Brain Tumor Study, Born 1978–1990.

Exposure				CasesN = 202 n (%)	Controls N = 285 n (%)	Adj^a^ OR	95% CI
Maternal smoking (N = 125 cases; 200 controls^b^)							
	No exposure to tobacco smoke during pregnancy			104 (83.2)	153 (76.5)	1.00	
		Mother smoked during pregnancy		21 (16.8)	47 (23.5)	0.55	0.29, 1.05
			Mother only	4 (3.2)	12 (6.0)	0.41	0.12, 1.42
			Mother and other passive/father^c^	17 (13.6)	35 (17.5)	0.60	0.30, 1.21
			1–10 cigarettes/day	5 (4.0)	26 (13.0)	0.23	0.08, 0.65
			11+ cigarettes/day	16 (12.8)	21 (10.5)	1.00	0.46, 2.17
			*P for trend*				0.42
Paternal smoking (N = 149 cases; 210 controls^d^)							
	No exposure to tobacco smoke during pregnancy			104 (69.8)	153 (72.9)	1.00	
		Father smoked during pregnancy		45 (30.2)	57 (27.1)	1.03	0.62, 1.71
			Father only	25 (16.8)	27 (12.9)	1.24	0.66, 2.35
			Father and other passive/mother^c^	20 (13.4)	30 (14.3)	0.82	0.41, 1.63
			≤3 hours/day^e^	24 (16.1)	33 (15.7)	0.86	0.46, 1.61
			>3 hours/day	21 (14.1)	24 (11.4)	1.30	0.65, 2.59
			*P for trend*				0.64

a Odds ratio and 95% CI, adjusted for race, sex, age at diagnosis/reference, mother’s education, birth year and center.

b Excludes children exposed to only paternal or other passive smoking.

c Other passive is exposure to tobacco smoke from a household resident other than the father, or at the workplace.

d Excludes children exposed to only maternal or other passive smoking.

e Hours per day of exposure from the father only, or from the father and another source.

We observed a statistically non-significant increased OR associated with paternal smoking during pregnancy (OR = 1.24; 95% CI: 0.66, 2.35). Exposure to paternal smoking for >3 hours per day, vs. no exposure, was positively associated with childhood brain tumors (OR = 1.30; 95% CI: 0.65, 2.59). The OR for smoking by both parents during pregnancy was consistent with no association (data not shown). Results were similar when examined by histology (data not shown). No association was observed for maternal exposure to tobacco smoke from other household residents. However, the number of mothers reporting exposure from other household members during pregnancy was small (10.4% of cases, 7.4% of controls; data not shown).

We modeled the direct genotype-childhood brain tumor association using ‘low-risk’ or ‘high-risk’ genotypes (see Table S1 in File S1). No polymorphisms were associated with childhood brain tumors.

When we examined the association between maternal and paternal smoking (never/ever during pregnancy) and childhood brain tumor risk, by ‘low-risk’ or ‘high-risk’ genotype, we found a positive interaction OR for paternal smoking and *EPHX1* H139R (OR_interaction_ = 2.21; *P*_interaction_ = 0.10, Table 4). In children with a high-risk genotype (HR/RR) for *EPHX1* H139R, exposure to paternal tobacco smoke during pregnancy was associated with increased risk of childhood brain tumors (OR = 1.78; 95% CI: 0.81, 3.91), whereas there was little observed association in children with a low-risk genotype (HH) (OR = 0.83; 95% CI: 0.45, 1.54). The case-only analysis showed a similar association (OR = 1.99; 95% CI: 0.96, 4.20; see Table S2 in File S1). Effect estimates changed minimally after adjustment for maternal smoking, with the exception of *SULT1A1* R213H: we found a statistically significantly increased OR for children with the high-risk genotype after adjustment (OR_high-risk_ = 2.19; 95% CI: 1.03, 4.65). Results were comparable when log-additive models were evaluated (data not shown). Other potential interactions were either statistically non-significant (e.g. mEH activity, *SULT1A1*, *GSTM1*) or did not manifest in a biologically plausible manner (e.g. *GSTP1* A114V) (see Table 4). We observed similar results for paternal smoking prior to pregnancy (never/ever) for all polymorphisms, with a positive interaction OR of a similar magnitude for *EPHX1* H139R (OR_interaction_ = 1.91; *P*_interaction_ = 0.13; data not shown). Results were similar when examined by histology (data not shown).

**Table 4:** Risk of Childhood Brain Tumors in Relation to Paternal Smoking during pregnancy by PAH Metabolism Genotype, West Coast Childhood Brain Tumor Study, Born 1978–1990.

Polymorphism		Low-risk genotype					High-risk genotype					Interaction OR^a^		*P*-value for interaction^a^	
		No/Yes	Adj.^b^OR	95% CI	Adj.^c^OR	95% CI	No/Yes	Adj.^b^OR	95% CI	Adj.^c^OR	95% CI	b	c	b	c
*EPHX1*H139R	Cases	107/24					44/21								
	Controls	144/37	0.83	0.45, 1.54	1.10	0.57, 2.11	82/20	1.78	0.81, 3.91	1.84	0.81, 4.21	2.21	2.26	0.10	0.10
Y113H	Cases	67/21					84/24								
	Controls	113/27	0.99	0.52, 1.89	1.17	0.60, 2.33	113/30	1.42	0.69, 2.94	1.54	0.72, 3.32	1.19	1.27	0.71	0.62
C-613T	Cases	70/26					81/19								
	Controls	126/35	1.20	0.64, 2.26	1.47	0.75, 2.90	100/22	1.02	0.49, 2.12	1.16	0.55, 2.49	0.83	0.78	0.69	0.61
mEH Activity^d^	Cases	66/15					85/30								
	Controls	84/23	0.84	0.38, 1.84	1.01	0.45, 2.33	142/34	1.43	0.78, 2.64	1.56	0.82, 2.98	1.67	1.82	0.29	0.23
*MPO*G-463A^e^	Cases	104/32					47/13								
	Controls	150/35	1.29	0.71, 2.32	1.48	0.80, 2.77	76/21	0.81	0.35, 1.89	1.07	0.44, 2.64	0.67	0.64	0.43	0.39
*SULT1A1* R213H	Cases	74/19					77/26								
	Controls	121/30	0.76	0.38, 1.54	0.85	0.41, 1.75	105/27	1.52	0.77, 3.00	2.19	1.03, 4.65	1.61	1.75	0.31	0.25
*NQO1*P187S	Cases	82/29					69/16								
	Controls	135/41	0.93	0.51, 1.69	1.08	0.57, 2.03	91/16	1.22	0.54, 2.75	1.50	0.63, 3.58	1.28	1.25	0.62	0.66
*GSTP1*I105V	Cases	66/17					85/28								
	Controls	74/18	1.01	0.46, 2.24	1.26	0.54, 2.98	152/39	1.10	0.60, 2.01	1.26	0.67, 2.37	1.16	1.10	0.77	0.85
A114V^e^	Cases	132/42					19/3								
	Controls	188/41	1.30	0.78, 2.18	1.66	0.95, 2.89	38/15	0.33	0.07, 1.57	0.32	0.06, 1.66	0.27	0.25	0.08	0.07
*GSTM1* f	Cases	82/20					68/25								
	Controls	109/30	0.74	0.37, 1.50	0.82	0.39, 1.72	117/27	1.46	0.75, 2.87	1.86	0.90, 3.83	1.88	1.81	0.18	0.22

a Interaction between genotype and smoking, using dichotomous genotype and exposure levels never and ever.

b Adjusted for race, sex, age at diagnosis, mother’s education, birth year and center.

c Additionally adjusted for maternal smoking.

d Microsomal epoxide hydrolase (mEH) activity: low–0,1 or 2 stable alleles (HH/HH, HH/HR, HY/HH, HH/RR, HY/HR, YY/HH); high–3 or 4 stable alleles (HY/RR, YY/HR, YY/RR).

e Missing gene information for 1 control.

f Missing gene information for 1 case.

As with paternal smoking, we observed an interaction between maternal smoking and *EPHX1* H139R (OR_interaction_ = 4.18; *P*_ interaction_ = 0.02; Table 5). Although shifted downward relative to paternal smoking ORs, the OR for children with a high-risk variant was again greater than that for children with a low-risk variant (*EPHX1* H139R: OR_high-risk_ = 1.09; 95% CI: 0.44, 2.71; OR_low-risk_ = 0.28; 95% CI: 0.12, 0.68). A similar interaction was observed for mEH activity (OR_high-risk_ = 0.87; 95% CI: 0.42, 1.79; OR_low-risk_ = 0.25; 95% CI: 0.07, 0.85; OR_interaction_ = 4.49; *P*_interaction_ = 0.03). The findings were supported by case-only analyses (*EPHX1* H139R: OR = 3.07; 95% CI: 1.14, 8.28; mEH activity: OR = 3.29; 95% CI: 1.01, 10.8; see Table S2 in File S1). Results were similar after adjustment for paternal smoking. Results did not differ by state (CA or WA) or histology (data not shown). Smaller and statistically non-significant positive interaction ORs were observed for *EPHX1* H139R and mEH activity for maternal smoking prior to pregnancy (never/ever).

**Table 5:** Risk of Childhood Brain Tumors in Relation to Maternal Smoking during pregnancy by PAH Metabolism Genotype, West Coast Childhood Brain Tumor Study, Born 1978–1990.

Polymorphism		Low-risk genotype					High-risk genotype					Interaction OR^a^		*P*-value for interaction^a^	
		No/Yes	Adj.^b^OR	95% CI	Adj.^c^OR	95% CI	No/Yes	Adj.^b^OR	95% CI	Adj.^c^OR	95% CI	b	c	b	c
*EPHX1* H139R	Cases	123/8					52/13								
	Controls	149/32	0.28	0.12, 0.68	0.27	0.11, 0.68	87/15	1.09	0.44, 2.71	0.88	0.34, 2.29	4.18	4.20	0.02	0.02
Y113H	Cases	99/9					76/12								
	Controls	119/24	0.46	0.19, 1.11	0.43	0.17, 1.09	117/23	0.85	0.36, 2.02	0.73	0.30, 1.81	1.96	1.98	0.26	0.25
C-613T	Cases	84/12					91/9								
	Controls	134/27	0.58	0.26, 1.28	0.49	0.21, 1.15	102/20	0.50	0.20, 1.30	0.48	0.18, 1.27	0.73	0.75	0.59	0.63
mEH Activity^d^	Cases	77/4					98/17								
	Controls	87/20	0.25	0.07, 0.85	0.25	0.07, 0.86	149/27	0.87	0.42, 1.79	0.74	0.34, 1.58	4.49	4.44	0.03	0.03
*MPO* G-463A^e^	Cases	120/16					55/5								
	Controls	156/29	0.66	0.32, 1.36	0.57	0.26, 1.22	80/17	0.25	0.08, 0.85	0.25	0.07, 0.87	0.59	0.60	0.42	0.44
*SULT1A1* R213H	Cases	82/11					93/10								
	Controls	131/20	0.56	0.22, 1.38	0.59	0.23, 1.50	105/27	0.41	0.17, 0.96	0.29	0.11, 0.74	0.48	0.47	0.21	0.20
*NQO1* P187S	Cases	96/15					79/6								
	Controls	144/32	0.56	0.27, 1.18	0.54	0.25, 1.19	92/15	0.47	0.16, 1.38	0.41	0.13, 1.25	0.74	0.74	0.63	0.64
*GSTP1* I105V	Cases	75/8					100/13								
	Controls	76/16	0.48	0.17, 1.40	0.43	0.14, 1.35	160/31	0.59	0.28, 1.25	0.54	0.25, 1.19	1.52	1.53	0.49	0.48
A114V^e^	Cases	155/19					20/2								
	Controls	188/41	0.49	0.26, 0.93	0.41	0.21, 0.80	47/6	0.70	0.09, 5.39	1.09	0.12, 10.1	1.19	1.14	0.85	0.89
*GSTM1* f	Cases	90/12					84/9								
	Controls	117/22	0.63	0.27, 1.45	0.68	0.28, 1.62	119/25	0.39	0.16, 0.98	0.32	0.13, 0.83	0.70	0.72	0.54	0.57

a Interaction between genotype and smoking, using dichotomous genotype and exposure levels never and ever.

b Adjusted for race, sex, age at diagnosis, mother’s education, birth year and center.

c Additionally adjusted for paternal smoking.

d Microsomal epoxide hydrolase (mEH) activity: low–0,1 or 2 stable alleles (HH/HH, HH/HR, HY/HH, HH/RR, HY/HR, YY/HH); high–3 or 4 stable alleles (HY/RR, YY/HR, YY/RR).

e Missing gene information for 1 control.

f Missing gene information for 1 case.

A positive association between hours per day of exposure to paternal smoking during pregnancy and childhood brain tumor risk was observed only among children with a high-risk genotype (HR or RR) for *EPHX1* H139R (*P*_interaction_ = 0.07; Table 6). For children with the high-risk genotype, those exposed to paternal smoking for >3 hours per day were 3.18 times as likely as unexposed children to develop a childhood brain tumor (95% CI: 0.92, 11.0). In contrast, among children with a low-risk genotype (HH), there was no childhood brain tumor-paternal smoking association (OR_ >3 hrs/day_ = 0.96; 95% CI: 0.42, 2.20). A similar association was seen for *SULT1A1* R213H, although the interaction did not reach statistical significance. Among children with a high-risk genotype (RR), children exposed to >3 hours per day of smoke from the father were 2.57 times as likely as unexposed children to develop a childhood brain tumor (95% CI: 0.94, 7.01). This association was greater after adjusting for maternal smoking during pregnancy (OR_ >3 hrs/day_ = 4.91; 95% CI: 1.55, 15.6; *P*_trend_ = 0.01). No increased risk was observed among children with a low-risk genotype (OR_ >3 hrs/day_ = 0.75; 95% CI: 0.28, 1.96). Adjustment for maternal smoking had minimal effects on remaining polymorphisms. A suggestion of increasing ORs among carriers of high-risk genotypes also was observed by duration of exposure for *EPHX1* Y113H and mEH activity (see Table 5), and for *NQO1* P187S (*P*_interaction_ = 0.54, data not shown).

**Table 6:** Risk of Childhood Brain Tumors in Relation to Paternal Smoking Level during pregnancy by Polymorphisms in Selected Genes, West Coast Childhood Brain Tumor Study, Born 1978–1990.

Polymorphism	Exposure^a^	Low-risk genotype					High-risk genotype						
		Cases/Controls	Adj.^b^ OR	95% CI	Adj.^c^ OR	95% CI	Cases/Controls	Adj.^b^ OR	95% CI	Adj.^c^OR	95% CI	*P*-value for interaction^d^	
												b	c
*EPHX1* H139R	Never	107/144	1.00		1.00		44/82	1.00		1.00		0.07	0.07
	≤3 hours	12/18	0.74	0.33, 1.65	0.83	0.36, 1.93	12/15	1.32	0.52, 3.34	1.37	0.53, 3.57		
	>3 hours	12/19	0.96	0.42, 2.20	1.54	0.62, 3.83	9/5	3.18	0.92, 11.0	3.32	0.93, 11.9		
*P for trend*				0.71		0.52			0.07		0.07		
*EPHX1* Y113H	Never	84/113	1.00		1.00		67/113	1.00		1.00		0.47	0.45
	≤3 hours	17/19	1.01	0.48, 2.14	1.23	0.80, 1.89	7/14	0.87	0.31, 2.48	0.93	0.32, 2.70		
	>3 hours	7/11	0.93	0.32, 2.73	1.4	0.91, 2.15	14/13	2.03	0.83, 4.99	2.31	0.89, 6.01		
*P for trend*				0.93		0.64			0.18		0.12		
mEH Activity^e^	Never	66/84	1.00		1.00		85/142	1.00		1.00		0.16	0.12
	≤3 hours	10/12	0.94	0.36, 2.44	1.11	0.42, 2.96	14/21	1.05	0.48, 2.29	1.13	0.51, 2.50		
	>3 hours	5/11	0.69	0.21, 2.31	0.88	0.25, 3.15	16/13	2.08	0.90, 4.79	2.41	0.98, 5.89		
*P for trend*				0.58		0.94			0.12		0.08		
*SULT1A1* R213H	Never	74/121	1.00		1.00		77/105	1.00		1.00		0.23	0.16
	≤3 hours	10/16	0.78	0.32, 1.92	0.84	0.34, 2.10	14/17	1.09	0.48, 2.48	1.44	0.60, 3.43		
	>3 hours	9/14	0.75	0.28, 1.96	0.86	0.32, 2.30	12/10	2.57	0.94, 7.01	4.91	1.55, 15.6		
*P for trend*				0.47		0.69			0.10		0.01		

a Hours of exposure per day.

b Adjusted for race, sex, age at diagnosis/reference, mother’s education, birth year and center.

c Additionally adjusted for maternal smoking.

d Interaction between genotype and smoking, using hours of exposure per day (interaction for trend).

e Microsomal epoxide hydrolase (mEH) activity: low–0,1 or 2 stable alleles (HH/HH, HH/HR, HY/HH, HH/RR, HY/HR, YY/HH); high–3 or 4 stable alleles (HY/RR, YY/HR, YY/RR).

Similar to the paternal smoking data, a statistically significant interaction was observed for level of maternal smoking during pregnancy and *EPHX1* H139R genotype (*P*_interaction_ = 0.003; see Table S3 in File S1). An interaction also was observed for mEH activity (*P*_interaction_ = 0.03). Among children with a high-risk variant (RR or HR) for *EPHX1* H139R, children whose mothers smoked 11 or more cigarettes per day were twice as likely to develop a childhood brain tumor as children of mothers who did not smoke (OR = 2.19; 95% CI: 0.72, 6.63), however, the number of children exposed to high levels of maternal smoking was low. Among children with a low-risk genotype, there was no increase in childhood brain tumor risk observed in relation to smoking. Results were similar for high mEH activity. Effect estimates decreased slightly after adjustment for paternal smoking.

**Figure 1:**
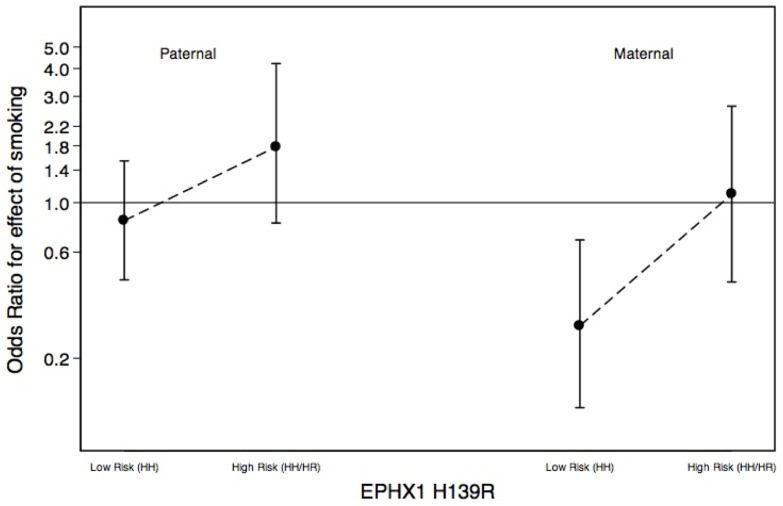
Risk of Childhood Brain Tumors by *EPHX1* H139R Genotype and Exposure to Parental Smoking (Maternal/Paternal), West Coast Childhood Brain Tumor Study, Born 1978–1990.

Figure 1 shows trends in childhood brain tumor risk by *EPHX1* H139R genotype for children exposed to paternal or maternal tobacco smoke during pregnancy. The pattern of increased risk associated with exposure to tobacco smoke for children with a high-risk genotype, in contrast to those with a low-risk genotype, persists for children exposed to either maternal or paternal smoking, as evidenced by the parallel interactions presented. Similar patterns were observed for mEH activity.

## Discussion

Our study expands on previous studies by evaluating the modifying effect of selected genetic polymorphisms involved in the metabolism of carcinogens present in tobacco smoke. We identified biologically plausible interactions between *EPHX1* H139R and both maternal and paternal smoking overall (never/ever) and by level of exposure. Our results suggest that childhood brain tumor risk may be associated with exposure to parental smoking during pregnancy for children with genetic susceptibility to carcinogenic PAHs present in tobacco smoke.

mEH is considered a detoxification enzyme for many substrates. However, in the process of PAH detoxification, carcinogenic highly-activated intermediates may be generated. mEH metabolizes PAHs to bay region diol-epoxides [26] that have potential to bind to DNA and cause mutations. A variant at exon 4 in *EPHX1* results in increased mEH activity [27], and presumably greater levels of activated PAHs. Further, *EPHX1* is expressed in the brain and during the fetal period [38].

The parental smoking-childhood brain tumor association was quite different for maternal vs. paternal smoking, overall and by strata of genotype for *EPHX1* H139R and mEH activity. In our primary associations analysis, exposure to maternal smoking during pregnancy resulted in an OR <1, whereas exposure to paternal smoking was positively associated with childhood brain tumors. However, interaction ORs for childhood brain tumors, *EPHX1* H139R and parental smoking were above null for both maternal smoking and paternal smoking (Figure 1). Similar results were observed for mEH activity.

Potential reasons for the observed protective association between maternal smoking (disregarding genotype) and childhood brain tumors are likely related to one of two different explanations. First, the data on maternal smoking during pregnancy may be subject to maternal reporting bias. If mothers of cases were more likely than mothers of controls to underreport smoking, an artificially low association could result. Second, a similar bias could have occurred if among smokers we contacted, mothers of cases were less likely than mothers of controls to participate in the study. The occurrence of ORs <1 for maternal smoking during pregnancy, especially more recently when smoking has become less socially acceptable, is consistent with either possible source of bias. Although these factors may have biased the maternal smoking-childhood brain tumor association downward, they are unlikely to account for the observed interactions. Gene-environment interactions are largely unaffected by selection bias [39] and biased conservatively by any reporting/recall that may differ by case status [40]. Confirmation of the interactions in the case-only analysis suggests the finding is not due to control selection or differential reporting.

The differences in maternal vs. paternal smoking ORs may be due to true biological differences in these associations with childhood brain tumor risk. However, if this were the case, we might have expected to observe dissimilar interaction ORs for maternal and paternal smoking with respect to *EPHX1* H139R genotype. Our data suggest that children with a high-risk genotype are at a greater risk of childhood brain tumors if exposed to either maternal or paternal smoking during pregnancy, relative to children with a low-risk genotype and similar exposures. This may indicate that PAH activation increases risk regardless of the source of parental exposure.

The carcinogenic process may be initiated through maternal exposure to environmental tobacco smoke from the father, or through the sperm, as a result of paternal smoking shortly before the child’s conception. Although our primary results focused on paternal smoking during pregnancy, we observed similar interaction ORs for paternal smoking prior to pregnancy. Paternal smoking may induce genotoxic effects on sperm; studies of male smokers have demonstrated greater levels of oxo^8^dG (an oxidative product of DNA damage) [41], 8-hydroxydeoxyguanosine [42], and benzo(a)pyrene diol epoxide-DNA adducts [43], [44] in sperm DNA, and an increased risk of aneuploidy [45]. However, the potential role of these in the etiology of brain tumors has not been established.

Both strengths and limitations of this analysis need to be considered in the interpretation of the data. Although this is a relatively large population-based study of childhood brain tumors with comprehensive ascertainment of cases and highly comparable population-based controls, our sample is small for gene-environment interaction analyses. Therefore, these findings could be explained by chance. We also focused on polymorphisms from a small number of candidate genes relevant to PAH specifically. We did not explore other genes associated with metabolism of other potential carcinogens in tobacco smoke and therefore may have missed some important interactions. We did not have DNA or genotype data for mothers, which during the pregnancy could influence PAH metabolism in combination with the child’s genotype. However, to our knowledge this is the first assessment of these interactions. Moreover, use of archival dried blood spots allowed inclusion of all cases regardless of survival status, therefore minimizing survival bias that may be problematic in case-control studies of highly fatal diseases.

Our study supports previous findings that parental smoking may be a risk factor for childhood brain tumors, and provides new information that risk may vary by genetic susceptibility. Studies that have reported no association may have been limited by inaccurate self-report of maternal smoking, and a lack of data on the genetic susceptibility of children in the study. Future studies of childhood brain tumors and parental smoking should include biological markers of smoking, in addition to data on the genetic susceptibility of children to tobacco smoke, to confirm and extend the results reported here.

## Supporting Information

File S1 **Table S1.** Risk of childhood brain tumors in relation to polycyclic aromatic hydrocarbon (PAH) metabolism polymorphisms, West Coast Childhood Brain Tumor Study, N = 479. **Table S2.** Association between exposure to prenatal parental smoking and selected polymorphisms in a case-only analysis, West Coast Childhood Brain Tumor Study, N = 196. **Table S3.** Risk of childhood brain tumors in relation to maternal smoking level during pregnancy by polymorphisms in selected genes, West Coast Childhood Brain Tumor Study.
(DOCX)
