# Supplementary material for: Parental Smoking and Risk of Childhood Brain Tumors by Functional Polymorphisms in Polycyclic Aromatic Hydrocarbon Metabolism Genes
Source: PLoS One. 2013 Nov 18;8(11):e79110. doi: 10.1371/journal.pone.0079110 (PMC3832498; doi:10.1371/journal.pone.0079110)
Supplement: File S1 — Table S1. Risk of childhood brain tumors in relation to polycyclic aromatic hydrocarbon (PAH) metabolism polymorphisms, West Coast Childhood Brain Tumor Study, N = 479. Table S2. Association between exposure to prenatal parental smoking and selected polymorphisms in a case-only analysis, West Coast Childhood Brain Tumor Study, N = 196. Table S3. Risk of childhood brain tumors in relation to maternal smoking level during pregnancy by polymorphisms in selected genes, West Coast Childhood Brain Tumor Study. (DOCX) [file pone.0079110.s001.docx]

**Supplemental Material**

Table of Contents

Supplemental Table 1. Risk of childhood brain tumors in relation to polycyclic aromatic hydrocarbon (PAH) metabolism polymorphisms, West Coast Childhood Brain Tumor Study, N=479 2

Supplemental Table 2. Association between exposure to prenatal parental smoking and selected polymorphisms in a case-only analysis, West Coast Childhood Brain Tumor Study, N=196 3

Supplemental Table 3. Risk of childhood brain tumors in relation to maternal smoking level during pregnancy by polymorphisms in selected genes, West Coast Childhood Brain Tumor Study 4

# Supplemental Table 1. Risk of childhood brain tumors in relation to polycyclic aromatic hydrocarbon (PAH) metabolism polymorphisms, West Coast Childhood Brain Tumor Study, N=479

| Gene | Polymorphism | Genotype | Cases | Controls | Adj . OR^a^ |  |
| --- | --- | --- | --- | --- | --- | --- |
|  |  |  | N(%) | N(%) |  |  |
|  |  |  | N=196 | N=283 |  | 95%CI |
| Microsomal expoxide hydrolase (mEH) (*EPHX1*) | | |  |  |  |  |
|  | H139R | HH (low-risk) | 131 (66.8) | 181 (64.0) | 1.00 |  |
|  |  | HR/RR (high-risk) | 65 (33.2) | 102 (36.0) | 1.15 | 0.64, 2.06 |
|  |  | |  |  |  |  |
|  | Y113H | HH/YH (low-risk) | 108 (55.1) | 143 (50.5) | 1.00 |  |
|  |  | YY (high-risk) | 88 (44.9) | 140 (49.5) | 1.15 | 0.65, 2.02 |
|  |  | |  |  |  |  |
|  | C-613T | CC (low-risk) | 96 (49.0) | 161 (56.9) | 1.00 |  |
|  |  | CT/TT (high-risk) | 100 (51.0) | 122 (43.1) | 0.74 | 0.42, 1.30 |
|  |  | |  |  |  |  |
|  | mEH Activity^b^ | Low (low-risk) | 81 (41.3) | 107 (37.8) | 1.00 |  |
|  |  | High (high-risk) | 115 (58.7) | 176 (62.2) | 0.97 | 0.55, 1.72 |
|  |  | |  |  |  |  |
| Myeloperoxidase (*MPO*) | |  |  |  |  |  |
|  | G-463A^c^ | AA (low-risk) | 136 (69.4) | 185 (65.6) | 1.00 |  |
|  |  | GA/GG (high-risk) | 60 (30.6) | 97 (34.4) | 1.11 | 0.61, 2.04 |
|  |  | |  |  |  |  |
| Sulfotransferase (*SULT1A1*) | |  |  |  |  |  |
|  | R213H | RH/HH (low-risk) | 93 (47.5) | 151 (53.4) | 1.00 |  |
|  |  | RR (high-risk) | 103 (52.5) | 132 (46.7) | 0.63 | 0.35, 1.12 |
|  |  | |  |  |  |  |
| NAD(P)H: quinone oxidoreductase (*NQO1*) | | |  |  |  |  |
|  | P187S | PP (low-risk) | 111 (56.6) | 176 (62.2) | 1.00 |  |
|  |  | PS/SS (high-risk) | 85 (43.4) | 107 (37.8) | 0.67 | 0.37, 1.23 |
|  |  | |  |  |  |  |
| Glutathione S, Transferase Pi 1 (*GSTP1*) | | |  |  |  |  |
|  | I105V | II (low-risk) | 83 (42.3) | 92 (32.5) | 1.00 |  |
|  |  | IV/VV (high-risk) | 113 (57.7) | 191 (67.5) | 0.76 | 0.42, 1.36 |
|  |  | |  |  |  |  |
|  | A114V^c^ | AA (low-risk) | 174 (88.8) | 229 (81.2) | 1.00 |  |
|  |  | AV/VV (high-risk) | 22 (11.2) | 53 (18.8) | 0.66 | 0.31, 1.41 |
|  |  | |  |  |  |  |
| Glutathione S-Transferase Mu 1 (*GSTM1*) | | |  |  |  |  |
|  | Null^d^ | No (low-risk) | 102 (52.3) | 139 (49.1) | 1.00 |  |
|  |  | Yes (high-risk) | 93 (47.8) | 144 (50.9) | 0.64 | 0.37, 1.12 |
| ^a^Adjusted for race, sex, age at diagnosis/reference, mother's education, birth year and center | | | | | | |
| ^b^Microsomal epoxide hydrolase (mEH) activity: low—0,1 or 2 stable alleles (HH/HH, HH/HR, HY/HH, HH/RR, HY/HR, YY/HH); high—3 or 4 stable alleles (HY/RR, YY/HR, YY/RR) | | | | | | |
| ^c^Missing gene information for 1 control | | | | | | |
| ^d^Missing gene information for 1 case | | | | | | |

# Supplemental Table 2. Association between exposure to prenatal parental smoking and selected polymorphisms in a case-only analysis, West Coast Childhood Brain Tumor Study, N=196

|  | Polymorphism | Genotype | Exposed N(%) | Unexposed N(%) | Adj. OR^a^ | 95% CI | Adj. OR^b^ | 95% CI |
| --- | --- | --- | --- | --- | --- | --- | --- | --- |
|  |  |  | N = 21 | N = 175 |  |  |  |  |
| *Paternal Smoking* | |  |  |  |  |  |  |  |
|  | EPHX1 H139R | HH | 24 (51.1) | 110 (71.0) | 1.00 |  | 1.00 |  |
|  |  | HR/RR | 23 (48.9) | 45 (29.0) | 1.99 | 0.96, 4.20 | 1.60 | 0.74, 3.47 |
|  |  |  |  |  |  |  |  |  |
|  | EPHX1 Y113H | YY | 21 (46.7) | 67 (44.4) | 1.00 |  | 1.00 |  |
|  |  | YH/HH | 24 (53.3) | 84 (55.6) | 1.18 | 0.58, 2.43 | 1.01 | 0.47, 2.19 |
|  |  |  |  |  |  |  |  |  |
|  | mEH Activity | Slow | 15 (31.9) | 68 (43.9) | 1.00 |  | 1.00 |  |
|  |  | Intermediate/Fast | 32 (68.1) | 87 (56.1) | 1.55 | 0.74, 3.25 | 1.24 | 0.57, 2.71 |
|  |  |  |  |  |  |  |  |  |
|  | SULT1A1 R213H | RH/HH | 19 (42.2) | 74 (49.0) | 1.00 |  | 1.00 |  |
|  |  | RR | 26 (57.8) | 77 (51.0) | 1.51 | 0.73, 3.11 | 1.56 | 0.72, 3.35 |
| *Maternal Smoking* | |  |  |  |  |  |  |  |
|  | EPHX1 H139R | HH | 8 (38.1) | 123 (70.3) | 1.00 |  | 1.00 |  |
|  |  | HR/RR | 13 (61.9) | 52 (29.7) | 3.07 | 1.14, 8.28 | 2.51 | 0.88, 7.16 |
|  |  |  |  |  |  |  |  |  |
|  | EPHX1 Y113H | YY | 12 (57.1) | 76 (43.4) | 1.00 |  | 1.00 |  |
|  |  | YH/HH | 9 (42.9) | 99 (56.6) | 1.88 | 0.70, 5.15 | 1.87 | 0.64, 5.44 |
|  |  |  |  |  |  |  |  |  |
|  | mEH Activity | Slow | 4 (19.1) | 77 (44.0) | 1.00 |  | 1.00 |  |
|  |  | Intermediate/Fast | 17 (80.9) | 98 (56.0) | 3.29 | 1.01, 10.8 | 2.99 | 0.87, 10.3 |
|  |  |  |  |  |  |  |  |  |
|  | SULT1A1 R213H | RH/HH | 11 (52.4) | 82 (46.9) | 1.00 |  | 1.00 |  |
|  |  | RR | 10 (47.6) | 93 (53.1) | 1.08 | 0.41, 2.85 | 0.89 | 0.31, 2.50 |
| ^a^Adjusted for race, sex, age at diagnosis, mother's education, birth year and center | | | | | | | | |
| ^b^Additionally adjusted for spousal smoking (maternal or paternal) | | | | | | | | |

# Supplemental Table 3. Risk of childhood brain tumors in relation to maternal smoking level during pregnancy by polymorphisms in selected genes, West Coast Childhood Brain Tumor Study

| Polymorphism | Exposure^a^ | Low-risk genotype | | | | | High-risk genotype | | | | |  |  |
| --- | --- | --- | --- | --- | --- | --- | --- | --- | --- | --- | --- | --- | --- |
|  |  | Cases/ Controls | Adj.^b^ OR | 95%CI | Adj.^c^ OR | 95%CI | Cases/ Controls | Adj.^b^ OR | 95%CI | Adj.^c^ OR | 95%CI | p-value for interaction^d^ | |
|  |  |  |  |  |  |  |  |  |  |  |  | ^b^ | ^c^ |
| *EPHX1* | Never | 123/149 | 1.00 |  | 1.00 |  | 52/87 | 1.00 |  | 1.00 |  | 0.003 | 0.01 |
| H139R | 1-10 /day | 3/18 | 0.17 | 0.05, 0.62 | 0.17 | 0.04, 0.62 | 2/8 | 0.28 | 0.05, 1.50 | 0.24 | 0.04, 1.29 |  |  |
|  | 11+ / day | 5/14 | 0.46 | 0.15, 1.41 | 0.45 | 0.14, 1.44 | 11/7 | 2.19 | 0.72, 6.63 | 1.74 | 0.55, 5.51 |  |  |
| *P for trend* |  |  |  | 0.02 |  | 0.02 |  |  | 0.38 |  | 0.63 |  |  |
|  |  |  |  |  |  |  |  |  |  |  |  |  |  |
| *EPHX1* | Never | 99/119 | 1.00 |  | 1.00 |  | 76/117 | 1.00 |  | 1.00 |  | 0.83 | 0.34 |
| Y113H | 1-10 /day | 2/14 | 0.16 | 0.03, 0.77 | 0.16 | 0.03, 0.76 | 3/12 | 0.41 | 0.10, 1.65 | 0.37 | 0.09, 1.52 |  |  |
|  | 11+ / day | 5/14 | 0.95 | 0.31, 2.88 | 0.91 | 0.29, 2.89 | 9/11 | 1.39 | 0.48, 4.03 | 1.17 | 0.38, 3.55 |  |  |
| *P for trend* |  |  |  | 0.37 |  | 0.32 |  |  | 0.87 |  | 0.89 |  |  |
|  |  |  |  |  |  |  |  |  |  |  |  |  |  |
| mEH Activity^e^ | Never | 77/87 | 1.00 |  | 1.00 |  | 98/149 | 1.00 |  | 1.00 |  | 0.03 | 0.04 |
|  | 1-10 /day | 1/11 | 0.09 | 0.01, 0.77 | 0.09 | 0.01, 0.77 | 4/15 | 0.36 | 0.11, 1.18 | 0.33 | 0.10, 1.08 |  |  |
|  | 11+ / day | 3/9 | 0.59 | 0.13, 2.65 | 0.59 | 0.13, 2.76 | 13/12 | 1.57 | 0.63, 3.87 | 1.31 | 0.51, 3.38 |  |  |
| *P for trend* |  |  |  | 0.11 |  | 0.12 |  |  | 0.74 |  | 0.96 |  |  |
|  |  |  |  |  |  |  |  |  |  |  |  |  |  |
| *SULT1A1* | Never | 82/131 | 1.00 |  | 1.00 |  | 93/105 | 1.00 |  | 1.00 |  | 0.18 | 0.17 |
| R213H | 1-10 /day | 2/13 | 0.21 | 0.04, 1.08 | 0.23 | 0.04, 1.15 | 3/13 | 0.22 | 0.06, 0.85 | 0.18 | 0.05, 0.72 |  |  |
|  | 11+ / day | 9/7 | 1.05 | 0.33, 3.37 | 1.13 | 0.34, 3.73 | 7/14 | 0.62 | 0.22, 1.73 | 0.42 | 0.13, 1.29 |  |  |
| *P for trend* |  |  |  | 0.51 |  | 0.63 |  |  | 0.13 |  | 0.04 |  |  |
| ^a^Number of cigarettes smoked per day | | | | | | | | | | | | | |
| ^b^Adjusted for race, sex, age at diagnosis/reference, mother's education, birth year and center | | | | | | | | | | | | | |
| ^c^Additionally adjusted for paternal smoking | | | | | | | | | | | | | |
| ^d^Interaction between genotype and smoking, using cigarettes per day (interaction for trend) | | | | | | | | | | | | | |
| ^e^Microsomal epoxide hydrolase (mEH) activity: low—0,1 or 2 stable alleles (HH/HH, HH/HR, HY/HH, HH/RR, HY/HR, YY/HH); high—3 or 4 stable alleles (HY/RR, YY/HR, YY/RR) | | | | | | | | | | | | | |
